# Nanooptical elements for visual verification

**DOI:** 10.1038/s41598-021-81950-w

**Published:** 2021-01-28

**Authors:** Alexander Goncharsky, Anton Goncharsky, Dmitry Melnik, Svyatoslav Durlevich

**Affiliations:** 1 Research Computer Center, M.V. Lomonosov Moscow State University, Leninskiye Gory, 1, Building 4, Moscow, Russia 119991; 2 Computer Holography Centre Ltd, Str.2, Proezd 4922, Zelenograd, Moscow, Russia 124460

**Keywords:** Optics and photonics, Applied optics, Displays

## Abstract

This paper focuses on the development of flat diffractive optical elements (DOEs) for protecting banknotes, documents, plastic cards, and securities against counterfeiting. A DOE is a flat diffractive element whose microrelief, when illuminated by white light, forms a visual image consisting of several symbols (digits or letters), which move across the optical element when tilted. The images formed by these elements are asymmetric with respect to the zero order. To form these images, the microrelief of a DOE must itself be asymmetric. The microrelief has a depth of ~ 0.3 microns and is shaped with an accuracy of ~ 10–15 nm using electron-beam lithography. The DOEs developed in this work are securely protected against counterfeiting and can be replicated hundreds of millions of times using standard equipment meant for the mass production of relief holograms.

## Introduction

The first flat optical elements were used to protect banknotes and plastic cards in the late 1980s. These optical security elements had the form of flat diffractive optical elements (DOEs) whose microreliefs, when illuminated by white light, formed images for visual control^1^. Experts in the technology of banknote production viewed this event as somewhat exotic. Banknotes in circulation must meet very stringent requirements, and the first incorporated optical elements hardly helped to increase the banknote lifetime^2^. An obvious advantage of these elements was that they improved the protection of banknotes against counterfeiting. This was especially important because the late 1980s was the period during which scanners and high-resolution printing facilities first appeared on the market.

At present, flat optical elements are used to protect banknotes, IDs, passports, plastic cards, and brands in all developed countries^3^. Hundreds of companies develop and manufacture optical security elements, and the total turnover of these companies amounts to several billions of dollars. The manufacturing technology of optical security elements can be subdivided into the development and production of the original and the technology of its mass replication^1^.

Over the past 30 years, the technology for the mass replication of optical security elements has experienced much development, allowing a single original security element to be replicated hundreds of millions of times, thereby substantially reducing the cost of these copies in large projects. This is an outstanding achievement that has provided the technical conditions to be met by modern equipment to allow security elements to be replicated as numerous copies, with the microreliefs of the copied elements reproducing very precisely that of the original^3,4^.

Currently, the weakest aspect of the technology for producing security elements is the synthesis of their originals. As a result of 30 years of development of relief security holograms, practically all companies produce originals using the same equipment based on laser radiation. The technology for the synthesis of the hologram originals is widely used and is not knowledge-intensive. Everything is being counterfeited and forged: passports, ID cards, etc., and the same is true for optical elements.

The optical security elements developed in this paper are based on the e-beam technology for the synthesis of the originals. The capabilities of modern e-beam technologies substantially exceed those of the optical methods used to form the microrelief. E-beam technology offers more than just a high resolution^5^. The main difference is that e-beam technology can be used to synthesize asymmetric microreliefs with an accuracy of 10–15 nm^6^.

Note that the first attempts to use e-beam technology to protect banknotes date back to the late 1980s. However, the first security elements made using e-beam technology consisted of binary diffraction gratings and were no different from optically recorded security elements^7^. E-beam technology is knowledge-intensive and expensive, and therefore, only a few published studies have used it to synthesize security elements^8–10^.

The first optical security elements with asymmetric microreliefs synthesized using e-beam lithography were proposed in Ref.^11^. The authors of this study developed a method for synthesizing DOEs that create the effect of switching between two 2D images. The 2D colour image seen by the observer at the 0° position loses its colour and becomes grey when the element is turned by 180°. To obtain this visual effect, the optical element must have an asymmetric microrelief. This optical element is impossible to forge using standard optical methods of origination. E-beam technology also allows the synthesis of flat optical elements for the formation of 3D images^12–14^.

In this paper, we present methods for synthesizing fundamentally novel DOEs containing an area with an asymmetric microrelief. When illuminated by white light from a point source, the microrelief of a DOE forms an image consisting of several symbols, letters, or digits. When the optical element is tilted, the images move across the area with the asymmetric microrelief. The depth of the microrelief is 0.3 microns. The microrelief has a multilevel structure. The microrelief is formed with an accuracy of 10–15 nm. The torch on a smartphone can ideally serve as the source of light. This element cannot be forged using optical methods for recording the originals.

## Results

### Some information about flat optical elements

In this paper, we propose a new flat optical element designed such that its phase function has the form of the sum of the phase functions of a flat optical element—the kinoform that forms the image when the optical element is illuminated by a point source and the given function *h*(*x*, *y*), which forms the image of the optical element in the scattered light. We show that this idea is viable and allows one to form various easily controllable features for visual control of the authenticity of optical elements.

The first optical elements were proposed by Fresnel back in the early nineteenth century^15^. The phase function of the Fresnel lens is well known: $\varphi_{1} \left( {x,y} \right) = C\frac{{x^{2} + y^{2} }}{{2f{ }}}$, where *f* is the distance to the focal plane, *C* is an arbitrary constant, and *x* and *y* are the Cartesian coordinates in the plane of the optical element. One can use an optical element with a saddle-shaped phase function $\varphi_{2} \left( {x,y} \right) = Cxy$ as a flat lens. Analogously, we refer to this element as a Fresnel lens with a saddle-shaped phase function.

In this study, we use the kinoforms introduced by Ref.^16,17^ to form the security features. A distinction is made between two kinds of kinoforms that differ by the type of their microrelief: binary and multilevel. The microrelief of binary kinoforms has two levels. The energy efficiency of binary kinoforms in the first diffraction order is less than 50%. Multilevel kinoforms form the same image as binary kinoforms but theoretically may have an efficiency of approximately 100%. Hence, a multilevel kinoform has maximal theoretical efficiency when used to form arbitrary 2D images. Kinoforms have another important property. Binary kinoforms have symmetric relief and are capable of forming only images symmetric with respect to the zero order. Multilevel kinoforms can form images that may be both symmetric and asymmetric with respect to the zero order.

**Figure 1:**
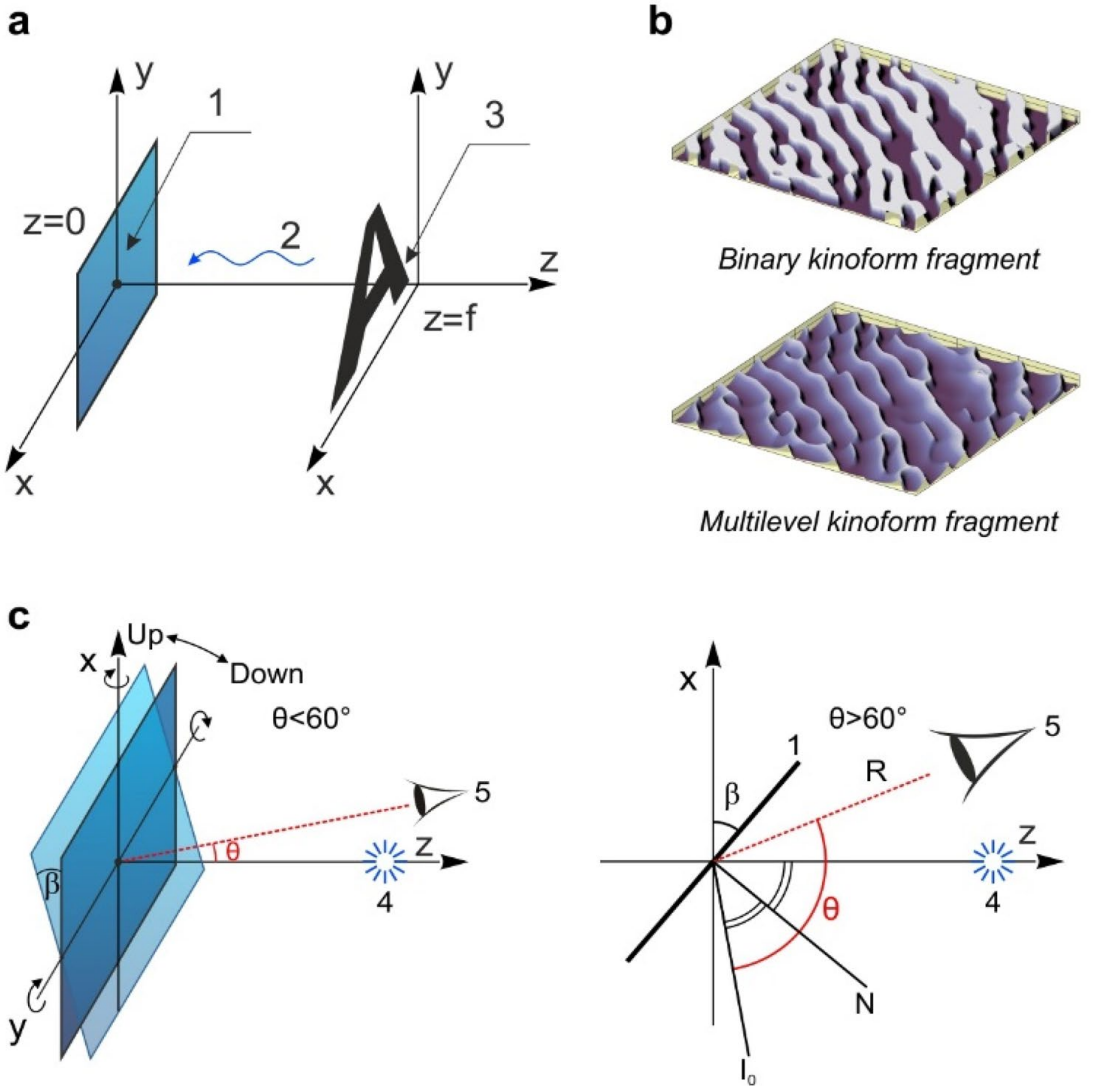
Formation of images using kinoforms. (**a**) Image formation scheme used to compute the phase function of the kinoform that forms the given image. (**b**) Fragments of a binary and a multilevel kinoform. (**c**) Observation schemes of the DOE at diffraction angles θ < 60° and θ > 60°.

Algorithms have been developed that make it possible to compute the phase function Φ(*x, y*) of the kinoform for the given properties of the source of light and of the image to be formed in the focal plane^17^. Figure 1a shows the scheme of image formation used to compute the phase function of the kinoform that forms the given image. Wave 2 from a point source is incident on flat optical element 1 located in the z = 0 plane. The image of symbol «A» forms in the z = f plane in the light scattered from the microrelief of the flat element. Figure 1b shows fragments of the microrelief of a multilevel and binary kinoform. The fragments have a size of 86 microns, and the maximum depth of the microrelief is approximately 0.3 microns.

Figure 1c shows the observation schemes of the optical element when it is illuminated by a point source at diffraction angles smaller and greater than 60°. The white-light point source is located on the Oz axis. The diffraction angle θ is the angle between the direction towards observer R and the direction towards the zero order of diffraction I_0_. The normal N is perpendicular to the plane of optical element 1. The optical element may turn about both the Ox axis (left/right) and the Oy axis (up/down). Hereafter, we denote the turn angle about the Ox and Oy axes by the letters γ and β, respectively.

### Design and methods for computing DOEs for the formation of full-parallax images

In this paper, we develop methods for the computer synthesis of DOEs forming 2D images with the effect of motion of several symbols inside selected area G of the optical element. The optical element has an asymmetric multilevel microrelief inside area G, and in the remaining area, the microrelief consists of binary diffraction gratings of different periods and different directions. The synthesis of images using diffraction gratings is a well-studied problem^18^ and is not a serious issue; thus, we concentrate on the problem of synthesizing a DOE in area G. To address this task, we need a special structure of the optical element. It seems appropriate to consider several variants of the structure of the optical element forming different types of kinematical motion of symbols inside selected area G. In this paragraph, we consider four variants of solving the synthesis problem, which differ in both the number of symbols and the pattern of their motion.

**Figure 2:**
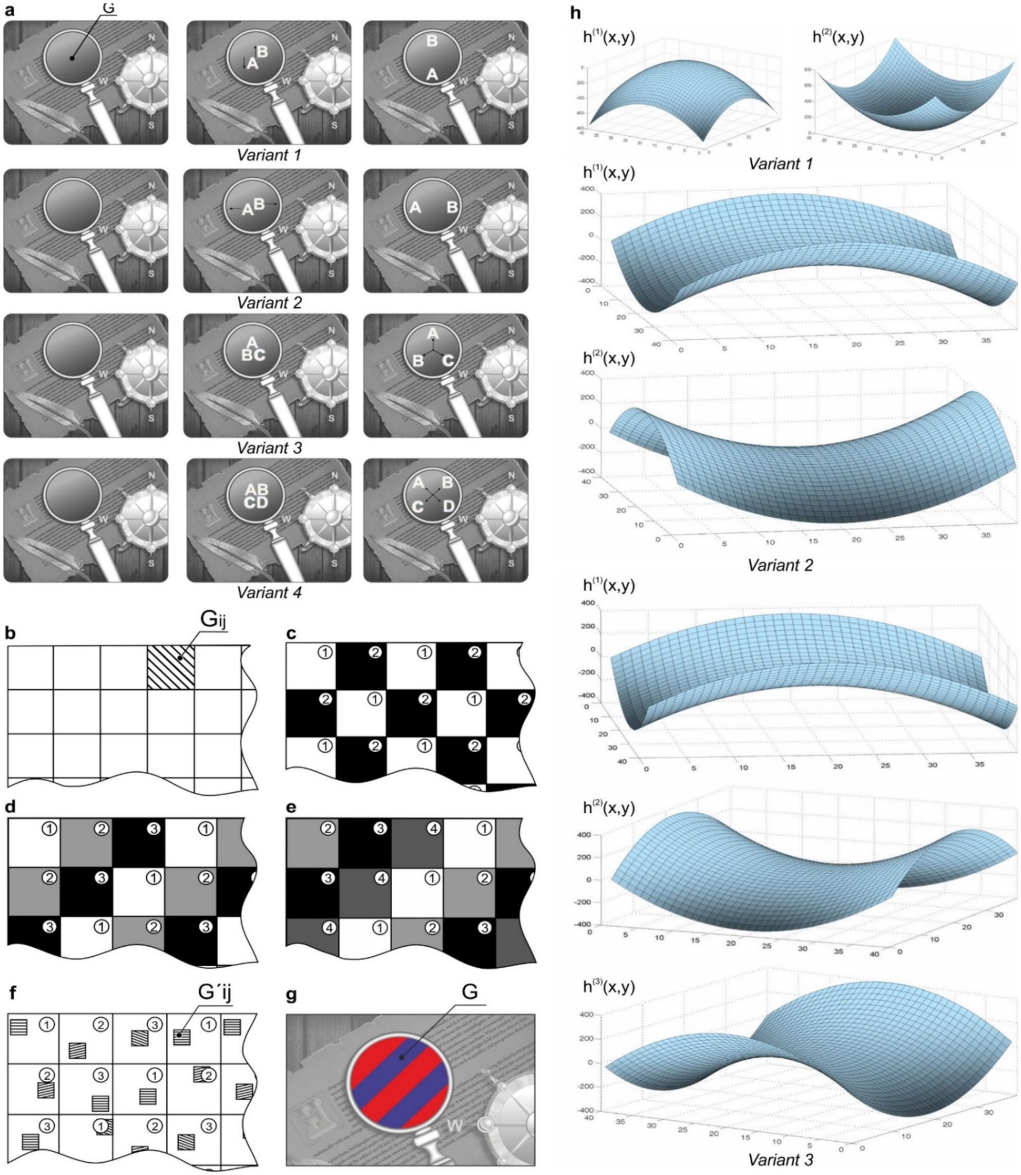
Design of the DOE in variants 1–4. (**a**) The design of the DOE when illuminated by scattered light (the left column) and the point source (the columns on the right). (**b**) The scheme of the partition of the DOE into elementary areas G_ij_. (**c**) The location map of two nonintersecting areas G^(1)^ and G^(2)^ in the area of optical element G. (**d**) The location map of three nonintersecting areas G^(1)^, G^(2)^, and G^(3)^ in the area of optical element G. (**e**) The location map of four nonintersecting areas G^(1)^, G^(2)^, G^(3)^, and G^(4)^ in the area of optical element G. (**f**) The location map of subareas ${\text{G}}_{{{\text{ij}}}}^{\prime }$ in elementary areas G_ij_. (**g**) Complementary colour image seen at diffraction angles greater than 60°. (**h**) Plots of the function h^(i)^(x, y) for variants 1–3.

We further assume that the source and position of the observer are fixed. When the optical element is illuminated by scattered light, the observer sees a uniform grey surface in selected area G of the optical element (Fig. 2a). Figure 2a also shows different variants of the formation of the images of several symbols seen by the observer when the optical element is illuminated by a white-light point source. To simplify the illustrations in Fig. 2a, we restrict the tilt of the DOEs to the up-down direction only (Fig. 1c). For each of these variants, the phase function can be written explicitly.

*In variant 1* shown in Fig. 2a, when the optical element is tilted up/down, symbols «A» and «B» also move up/down in opposite directions. The DOE has the form of a single-layer optical element located on a flat basis. Let us place the origin of the Cartesian coordinate system O*xyz* at the centre of area G. Let us subdivide area G into elementary areas G_ij_, i = 1, 2, … I, j = 1, 2, … J, with sizes no greater than 100 microns (Fig. 2b). Let us consider area G^(1)^ (Fig. 2c), which consists of elementary areas G_ij_, where (i + j) is even, and area G^(2)^, which consists of elementary areas G_ij_, where (i + j) is odd.

In the elementary areas G_ij_ belonging to G^(1)^, the phase function of the optical element is equal to the sum of the given function ${\text{h}}_{{{\text{ij}}}}^{(1)}$ (*x, y*) = C · ρ^2^ in the polar coordinate system *x* = ρ · cos φ, *y* = ρ · sin φ, and the phase function $\Phi_{{{\text{ij}}}}^{(1)} (x,y)$ of the multilevel kinoform that forms the image of symbol «A» in the focal plane z = const. In area G^(2)^, the phase function of the optical element is equal to the sum of the given function ${\text{h}}_{{{\text{ij}}}}^{(2)} \left( {x,y} \right)$ = − C · ρ^2^ and the phase function $\Phi_{{{\text{ij}}}}^{(2)} (x,y)$ of the multilevel kinoform that forms the image of symbol «B» in the focal plane z = const. When the optical element is tilted up/down, the symbols «A» and «B» also move in the vertical direction. The shapes of the surfaces ${\text{h}}_{{{\text{ij}}}}^{(1)} (x,y)$ and ${\text{h}}_{{{\text{ij}}}}^{(2)} \left( {x,y} \right)$ that define this type of movement are shown in Fig. 2h.

*In variant 2*, a structure can be proposed for the DOE to ensure a different pattern of motion of the symbols when the optical element is tilted. For example, when the element is tilted up/down, the symbols «A» and «B» shift left/right. Other variants of surfaces ${\text{h}}_{{{\text{ij}}}}^{(1)} (x,y)$ and ${\text{h}}_{{{\text{ij}}}}^{(2)} \left( {x,y} \right)$ must be used to synthesize this element. In variant 2, the phase function of the optical element for elementary areas G_ij_ belonging to G^(1)^ is equal to the sum of the given function ${\text{h}}_{{{\text{ij}}}}^{(1)} (x,y)$ = C · ρ^2^ cos(2φ) and the phase function $\Phi_{{{\text{ij}}}}^{(1)} (x,y)$ of the multilevel kinoform that forms the image of symbol «A». In elementary areas G_ij_ in area G^(2)^, the phase function of the optical element is equal to the sum of the given function ${\text{h}}_{{{\text{ij}}}}^{(2)} \left( {x,y} \right)$ = C · ρ^2^ sin(2φ) and the phase function $\Phi_{{{\text{ij}}}}^{(2)} (x,y)$ of the multilevel kinoform that forms the image of symbol «B». The shapes of the surfaces ${\text{h}}_{{{\text{ij}}}}^{(1)} (x,y)$ and ${\text{h}}_{{{\text{ij}}}}^{(2)} \left( {x,y} \right)$ for variant 2 are shown in Fig. 2h.

*In variant 3*, when the optical element is illuminated by a point source, the observer sees three symbols «A», «B», and «C», which «diverge away from each other» when the element is tilted, and the angles between the symbols are equal to 120°. Similar to variants 1 and 2, area G is subdivided into nonintersecting areas G^(1)^, G^(2)^, and G^(3)^, as shown in Fig. 2d. Area G^(1)^ consists of elementary areas G_ij,_ G^(2)^, and G^(3)^, denoted by digits **1**, **2**, and **3**, respectively. In elementary areas G_ij_ in area G^(1)^, the phase function of the optical element is equal to the sum of the given function ${\text{h}}_{{{\text{ij}}}}^{(1)} (x,y)$ = C · ρ^2^ cos(2φ) and the phase function of the kinoform $\Phi_{{{\text{ij}}}}^{(1)} (x,y)$ that forms the image of symbol «A». In elementary areas G_ij_ in area G^(2)^, the phase function of the optical element is equal to the sum of the given function ${\text{h}}_{{{\text{ij}}}}^{(2)} \left( {x,y} \right)$ = C · ρ^2^ cos(2 · (φ + π/3)) and the phase function $\Phi_{{{\text{ij}}}}^{(2)} (x,y)$ of the kinoform that forms the image of symbol «B». In elementary areas G_ij_ in area G^(3)^, the phase function of the optical element is equal to the sum of the given function ${\text{h}}_{{{\text{ij}}}}^{(3)} \left( {x,y} \right)$ = C · ρ^2^ cos(2 · (φ–π/3)) and the phase function $\Phi_{{{\text{ij}}}}^{(3)} (x,y)$ of the multilevel kinoform that forms the image of symbol «C». The shapes of the surfaces ${\text{h}}_{{{\text{ij}}}}^{(1)} (x,y)$, ${\text{h}}_{{{\text{ij}}}}^{(2)} \left( {x,y} \right)$, and ${\text{h}}_{{{\text{ij}}}}^{(3)} \left( {x,y} \right)$ for variant 3 are shown in Fig. 2h.

*In variant 4*, we present a DOE where the observer sees four symbols «A», «B», «C», and «D», which move across the area of the optical element when it is illuminated by a point source. Similar to variants 1 and 2, area G is subdivided into nonintersecting areas G^(1)^, G^(2)^, G^(3)^, and G^(4)^, as shown in Fig. 2e. In elementary areas G_ij_ in area G^(1)^, the phase function of the optical element is equal to the sum of the given function ${\text{h}}_{{{\text{ij}}}}^{(1)} (x,y)$ = C · ρ^2^ cos(2φ) and the phase function $\Phi_{{{\text{ij}}}}^{(1)} (x,y)$ of the multilevel kinoform that forms the image of symbol «A». In elementary areas G_ij_ in area G^(2)^, the phase function of the optical element is equal to the sum of the given function ${\text{h}}_{{{\text{ij}}}}^{(2)} \left( {x,y} \right)$ = C · ρ^2^ cos(2 · (φ + π/4)) and the phase function $\Phi_{{{\text{ij}}}}^{(2)} (x,y)$ of the multilevel kinoform that forms the image of symbol «B». In elementary areas G_ij_ in area G^(3)^, the phase function of the optical element is equal to the sum of the given function ${\text{h}}_{{{\text{ij}}}}^{(3)} \left( {x,y} \right)$ = C · ρ^2^ cos(2 · (φ + π/2)) and the phase function $\Phi_{{{\text{ij}}}}^{(3)} (x,y)$ of the multilevel kinoform that forms the image of symbol «C». In elementary areas G_ij_ in area G^(3)^, the phase function of the optical element is equal to the sum of the given function ${\text{h}}_{{{\text{ij}}}}^{(4)} \left( {x,y} \right)$ = C · ρ^2^ cos(2 · (φ + 3π/4)) and the phase function $\Phi_{{{\text{ij}}}}^{(4)} \left( {x,y} \right)$ of the multilevel kinoform that forms the image of symbol «D».

*In variant 4*, when the DOE is illuminated by a point source, the observer sees simultaneously the symbols «A», «B», «C», and «D». When the optical element is tilted, the symbols «A», «B», «C», and «D» move across the entire area of the optical element along four rays π/2 apart.

In variants 1–4, the observer sees the images of the symbols at diffraction angles less than 60°. The structure of the optical element can be upgraded so that at diffraction angles greater than 60°, the observer sees another colour image in area G. Let us select in each elementary area G_ij_ subarea G′_ij_, as shown in Fig. 2f. Subareas G′_ij_ can be filled with diffraction gratings with different directions and periods in the 0.4–0.5 micron interval such that at diffraction angles greater than 60°, the observer sees the image shown in Fig. 2g.

Computation of the phase function Φ(*x*, *y*) of the optical element that forms the given image F(*x*, *y*) is the classic inverse problem of flat optics. The inverse problem reduces to determining function Φ(*x*, *y*) from operator Eq. (1):

1 $$A{\Phi }\left( {\xi ,\eta } \right) = F\left( {x,y} \right).$$

In Eq. (1), $A{\Phi }$, Q, and F are defined by the following formulas:

2 $$A\Phi = \left| {\left. {\gamma \iint\limits_{{G_{ij} }} {\overline{u}\left( {\xi ,\eta ,} \right)\exp \left( {ik\Phi \left( {\xi ,\eta } \right)} \right)\exp \left\{ {ikQ\left( {\xi ,\eta ,x,y} \right)} \right\}d\xi d\eta }} \right|} \right.,$$

3 $$Q\left( {\xi ,\eta ,x,y} \right) = \frac{{\left( {x - \xi } \right)^{2} + \left( {y - \eta } \right)^{2} }}{2f},$$

4 $$\left| {u\left( {x,y,f} \right)} \right| = F\left( {x,y} \right).$$

Here, $\overline{u}\left( {\xi , \eta } \right)$ is the complex amplitude of the wave field incident on the optical element in the plane of the optical element; $f$ is the distance between the optical element and the image plane; and $u\left( {x,y,f} \right)$ is the amplitude of the wave field in the image plane. Equation (1) is a nonlinear Fredholm operator equation of the first kind. This is a so-called ill-posed problem^19,20^, and the methods for solving it were developed in the late twentieth century. Approximate solutions to problem (1) can be computed by minimizing the functional R(Φ) = ${\text{A}}\Phi - {\text{ F}}^{2}$. The functional R(Φ) is differentiable, and its gradient can be easily computed^21^. Functional R(Φ) can be minimized using iterative methods, e.g., gradient algorithms^22,23^. There are other iterative schemes for solving Eq. (1). In this paper, we use the method proposed by Lesem^17^ to compute the phase function. A detailed description of the iterative algorithm employed can be found in^11,24^. We can specify different images of the symbols «A», «B», «C», and «D» and compute the corresponding phase functions $\Phi_{{{\text{ij}}}}^{(1)} (x,y)$, $\Phi_{{{\text{ij}}}}^{(2)} (x,y)$, $\Phi_{{{\text{ij}}}}^{(3)} (x,y)$, and $\Phi_{{{\text{ij}}}}^{(4)} \left( {x,y} \right)$ in each elementary area G_ij_.

In the case of multilevel kinoforms, the phase function Φ(*x*, *y*) uniquely determines the depth of the microrelief. For reflecting optical elements with light normally incident onto the optical element, the microrelief depth at each point (*x, y*) is equal to 0.5·Φ(*x*, *y*)^11^.

**Figure 3:**
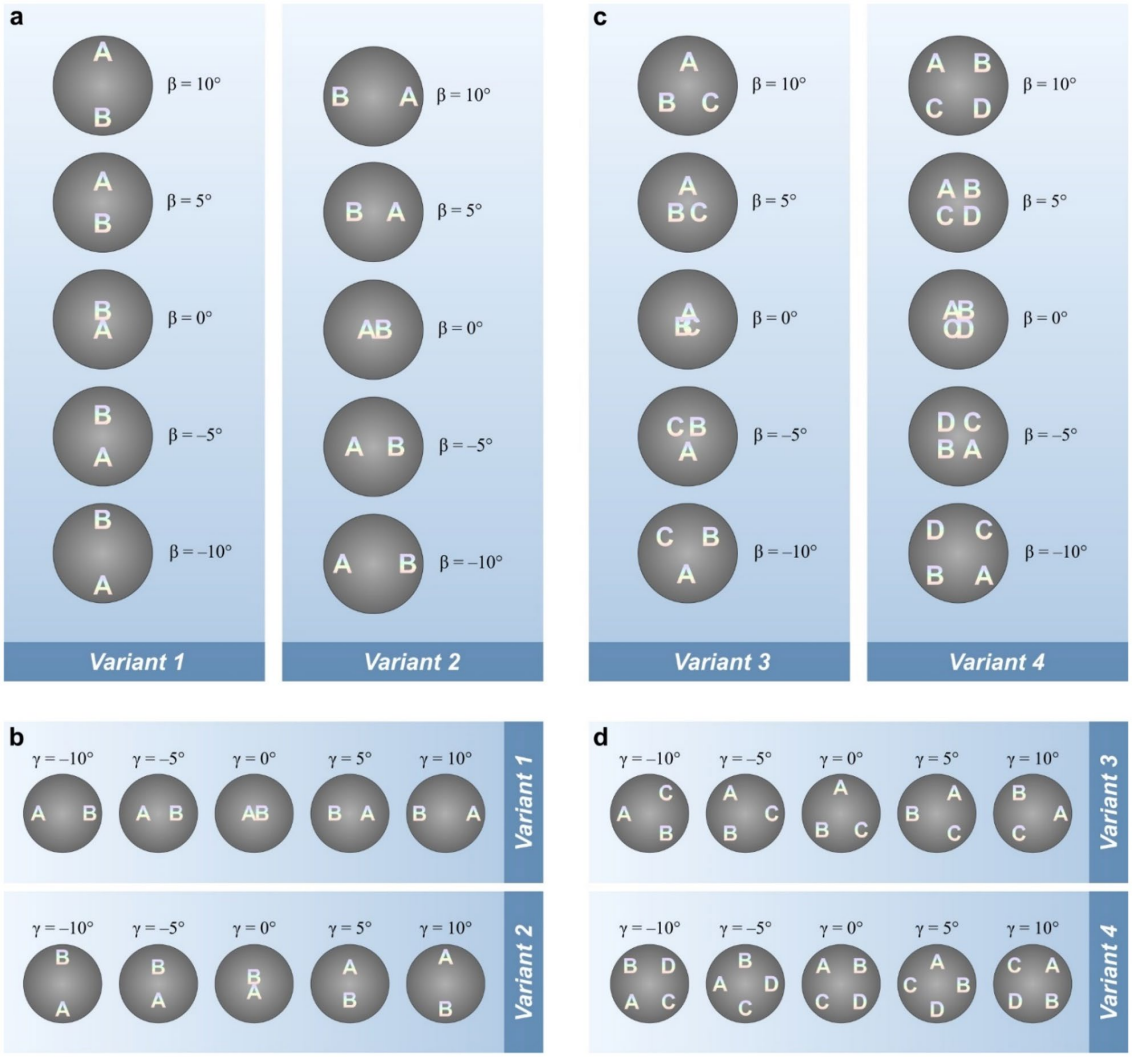
Kinematic effects of the motion of symbols in DOEs made according to variants 1–4. (**a**), Variants 1 and 2 in the case where the image is tilted up/down. (**b**) Variants 1 and 2 in the case where the image is tilted left/right. (**c**) Variants 3 and 4 in the case where the image is tilted up/down. (**b**) Variants 3 and 4 in the case where the image is tilted left/right.

Figure 3 illustrates the kinematic effect of the motion of symbols in variants 1–4 when the optical element is illuminated by a white-light source. Figure 3a shows the kinematic effects of the motion of two letters «A» and «B» in variants 1 and 2. When the optical element is tilted up/down, the symbols «A» and «B» also move up/down in variant 1 and left/right in variant 2.

In Fig. 3b, the optical element is tilted left/right. In variant 1, the symbols «A» and «B» also move left/right, whereas in variant 2, the symbols move up/down.

Figure 3c illustrates the kinematic motion of the symbols in variants 3 and 4. The optical element is tilted up/down, and parameter γ = 0. As is evident from Fig. 3, when the DOE is tilted up/down, the letters «A», «B», and «C» diverge away from the centre along three rays 120° apart. In variant 4, when the DOE is tilted up/down, the letters «A», «B», «C», and «D» diverge away from the centre along four rays 90° apart.

Figure 3d shows the images formed by the DOE in variants 3 and 4 when the optical system is tilted left/right. Angle γ varies, and angle β remains fixed and differs from zero. In variants 3 and 4, when the DOE is tilted left/right, the symbols rotate clockwise/anticlockwise with respect to the centre. The central frames correspond to the case when angle γ = 0.

The capabilities of the proposed synthesis technology go far beyond the variants presented above.

### Creation of sample optical security elements

**Figure 4:**
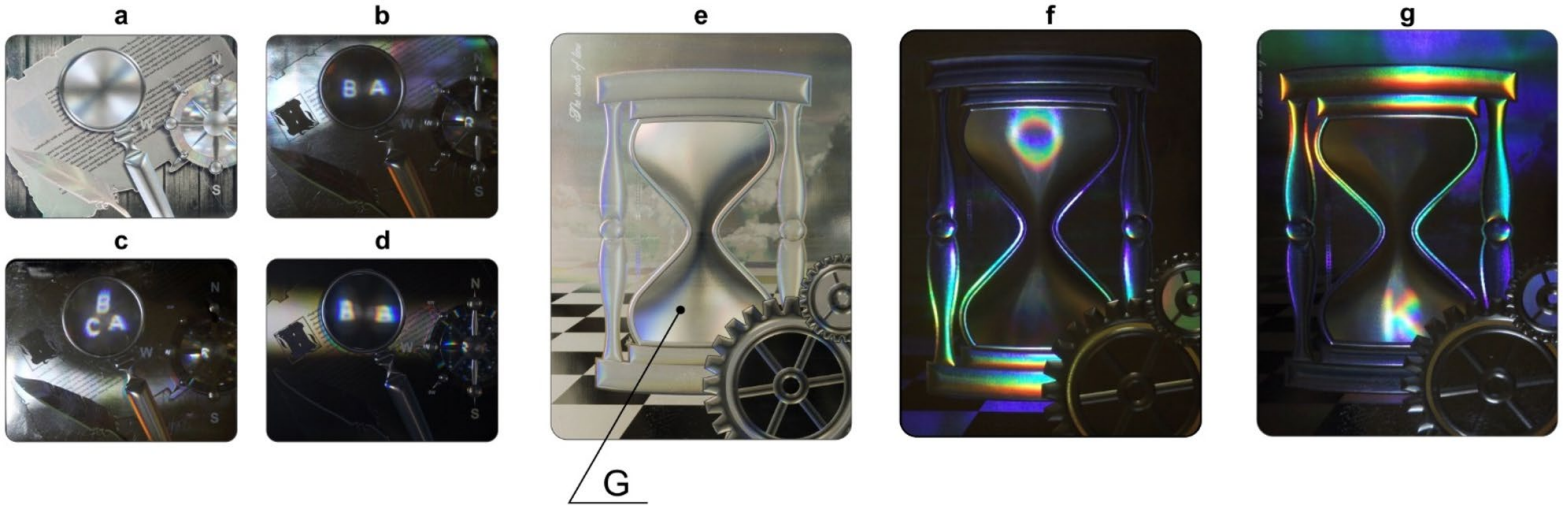
Photos of the manufactured DOEs. (**a**) Photo of a DOE (variant 1) taken in scattered light. (**b**) Photo of a DOE (variant 3) taken when illuminated by a point source (see Supplementary Video 1). (**c**) Photo of a DOE (variant 3) taken when illuminated by a point source (see Supplementary Video 2). (**d**) Photo of a DOE with a binary microrelief in variant 1 when the element is illuminated by a point source. (**e**) Photo of a DOE with an hourglass image when illuminated by scattered light. (**f**,**g**) Photos of a DOE taken at different tilt angles of the element when illuminated by a point source (see Supplementary Video 3).

To demonstrate the efficiency of the methods developed in this paper, we used e-beam technology to make three DOEs. We made the first two elements with the phase function presented in variants 1 and 3. Figure 4a shows a photo of DOEs illuminated by scattered light. In the photo, area G with a multilevel microrelief appears as the lens of a magnifying glass. Figure 4b–d show photos obtained when the element is illuminated by a point source. Figure 4b shows the photo of a DOE in variant 1 when the element is tilted left/right. When illuminated by a point source, the DOE forms an image consisting of two symbols «A» and «B». Figure 4c shows the photo of a DOE in variant 3. The image consists of three symbols «A», «B», and «C». Figure 4d shows the photo of a DOE with the binary phase function made in variant 1. As is evident from Fig. 4d, the symbols «A» and «B» are not identified. This means that the DOE cannot be forged with the widely used optical methods of origination^1,25^.

We developed the third DOE with an hourglass image to demonstrate the capabilities of our synthesis methods. The selected area G with asymmetric relief has the shape of an hourglass bulb (Fig. 4e). The remaining part of the optical elements has a symmetric relief and is formed using binary structures. When the optical element is illuminated by scattered light, area G appears as an achromatic bas-relief (Fig. 4e). When the optical element is illuminated by a point source, the observer sees at the surface of the bas-relief the symbols «O» and «К», which shift when the optical element is tilted. Figures 4f,g show two photos made at different up/down tilt angles of the DOE. As the letter «O» moves towards the centre of the hourglass, its size decreases along the horizontal direction; when it enters the lower part of the hourglass, the letter «O» transforms into the letter «К». The optical elements in Figs. 4a,b have a size of 42 × 33.6 mm^2^. The optical elements in Fig. 4e–g have a size of 37 × 50 mm^2^.

The microrelief of the optical elements was formed using a shaped e-beam lithography system with a minimum pixel size of 0.1 × 0.1 µm^2^. The maximum pixel size of the system may reach 6.3 × 6.3 µm^2^. The microrelief was shaped with an accuracy within 15 nm in height^6,26^ using a positive electron resist^5^. We used standard equipment for the production of relief security holograms to make small sets (5000 copies) of identical DOEs. The photos shown in Fig. 4 are of these mass-produced elements. The patterns of the motion of symbols when the optical element is tilted are demonstrated in Supplementary Videos 1–3.

### Discussion

In this paper, we analyse methods for the synthesis of DOEs based on the electron-beam technology of microrelief formation. The authenticity of the optical element is visually controlled. The DOE has an area G with a multilevel relief, which appears as a bas-relief when observed in scattered light. When the DOE is illuminated by a point source of white light, images of one or several symbols appear at the surface of the bas-relief. The optical element is illuminated by a white-light point source, ideally by the torch of a smartphone. The visual security feature is easy to control. The symbols «A», «B», and «C» can be made sharper by using a laser diode as the point source of light.

The accuracy in shaping the asymmetrical microrelief in the DOEs developed is 15 nm in height. These DOEs cannot be forged using common optical technologies of origination such as, e.g., dot-matrix technology^25^. It is impossible to identify the symbols on copies of the DOE made using these technologies.

The technology of synthesis of nanooptical security elements is based on electron-beam lithography of precision formation of microrelief and methods developed by the authors for solving nonlinear inverse problems of synthesis of flat optical elements. The methods for the synthesis of DOEs developed in this study allow the production of optical elements for protecting banknotes, excise stamps, IDs, passports, plastic cards, and brands to be taken to a new higher level.

Currently, many of the published studies used e-beam lithography to shape nanostructures in flat optical elements. The characteristic sizes of these structures may amount to several dozens of nanometres^27–29^. These studies are also of great interest for the development of security technologies, primarily those meant for shaping optical elements to be observed in transmitted light^30,31^. However, for the practical use of these optical elements, we still have to address the problem of their mass replication.

In this paper, we propose methods for the synthesis of DOEs allowing mass replication. The DOEs presented in this paper are already used to protect IDs, documents, and brands replicated millions of times against counterfeiting.

## Methods

### Design of the DOEs

The artwork was prepared using the Adobe Illustrator vector graphic editor. Several MATLAB scripts were written to compute the phase functions of the DOEs. The computations were carried out on a PC with an AMD Phenom II X6 3.2 GHz CPU and 16 GB of DDR3 memory without a GPU. The exposure data were prepared using the proprietary software of the e-beam lithography system. The total computational time for the exposure data of area G of the multilevel kinoform for each DOE was approximately 1 h.

### E-beam lithography

The exposure was performed on a glass plate with a positive-tone PMMA e-beam resist using a shaped-beam Carl Zeiss ZBA-21 lithography system with an accelerating voltage of 20 kV. The exposure time of area G of the multilevel kinoform was approximately 6.5 h for each DOE. The resist was developed with a 1:3 MIBK/IPA solution. The microrelief depth of the final DOE produced was 270 nm, and its shaping precision at depth was 15 nm.

### Mass replication

The plate with the developed e-beam resist was coated with silver using a vacuum evaporator VUP 2 M. To fabricate a copy of the microrelief on the metal plate, a galvanic process was used. Mass replication was performed using standard equipment for the step-repeat and embossing processes. Approximately 5000 copies were produced on a 40 micron thick PET-based self-adhesive holographic film with an aluminium coating.

### Shooting of photos and videos

All photos and videos were captured from the holographic film samples by a Sony Alpha A7 camera. The torch of a mobile phone was used as a spotlight source. The photos involving scattered light were taken in a room illuminated by several fluorescent lamps.

## Supplementary Information

Supplementary Information 1.

Supplementary Video 1.

Supplementary Video 2.

Supplementary Video 3.

## References

[1] Van Renesse RL (2005). Optical Document Security. Artech House optoelectronics library.

[2] Lancaster IM, van Renesse RL (2006). Use and efficacy of DOVIDs and other optical security devices. Optical Security and Counterfeit Deterrence Techniques VI, vol 6075, International Society for Optics and Photonics.

[3] Lancaster IM, Mitchell A, van Renesse RL (2004). The growth of optically variable features on banknotes. Optical Security and Counterfeit Deterrence Techniques V.

[4] Goncharsky AV, Goncharsky AA (2004). Computer Optics and Computer Holography.

[5] Rai-Choudhury P (1997). Handbook of Microlithography, Micromachining, and Microfabrication: Microlithography.

[6] Chen Y (2015). Nanofabrication by electron beam lithography and its applications: a review. Microelectron. Eng..

[7] Lee RA, Fagan WF (1991). Pixelgram: an application of electron-beam lithography for the security printing industry. Holographic Optical Security Systems, vol 1509.

[8] Firsov A (2014). Fabrication of digital rainbow holograms and 3-d imaging using SEM based e-beam lithography. Opt. Express.

[9] Verheijen M (1993). E-beam lithography for digital holograms. J. Mod. Opt..

[10] Ruffato G (2017). Design, fabrication and characterization of computer generated holograms for anti-counterfeiting applications using OAM beams as light decoders. Sci. Rep..

[11] Goncharsky AV, Goncharsky AA, Durlevich S (2015). Diffractive optical element with asymmetric microrelief for creating visual security features. Opt. Express.

[12] Goncharsky AA, Durlevich S (2018). Cylindrical computer-generated hologram for displaying 3d images. Opt. Express.

[13] Goncharsky A, Goncharsky A, Durlevich S (2016). Diffractive optical element for creating visual 3d images. Opt. Express.

[14] Goncharsky AV, Goncharsky AA, Durlevich S (2017). High-resolution full-parallax computer-generated holographic stereogram created by e-beam technology. Opt. Eng..

[15] Fresnel A (1816). Memoir on the diffraction of light. Annal. Chim..

[16] Hirsch, P. M., Jordan, J. A. & Lesem, L. B. Method of making an object dependent diffuser (1971). U.S. Patent 3,619,022.

[17] Lesem LB, Hirsch PM, Jordan JA (1969). The kinoform: a new wavefront reconstruction device. IBM J. Res. Dev..

[18] Palmer C (2005). Diffraction Grating Handbook.

[19] Tikhonov AN (1963). The solution of ill-posed problems and the regularization method. Dokl. Akad. Nauk SSSR.

[20] Tikhonov AN, Goncharsky AV, Stepanov VV, Yagola AG (1995). Numerical Methods for the Solution of Ill-Posed Problems.

[21] Kolmogorov A, Fomin S (1996). Elements of the Theory of Functions and Functional Analysis: Metric and Normed Spaces.

[22] Bakushinsky A, Goncharsky A (1994). Ill-Posed Problems: Theory and Applications.

[23] Nemirovskij A, Polyak B (1984). Iterative methods for solving linear ill-posed problems under precise information. Eng. Cybern..

[24] Gerchberg RW, Saxton WO (1972). A practical algorithm for the determination of the phase from image and diffraction plane pictures. Optik.

[25] Tamulevicius T (2018). Dot-matrix hologram rendering algorithm and its validation through direct laser interference patterning. Sci. Rep..

[26] Ekberg M, Nikolajeff F, Larsson M, Hård S (1994). Proximity-compensated blazed transmission grating manufacture with direct-writing, electron-beam lithography. Appl. Opt..

[27] Goh XM (2014). Three-dimensional plasmonic stereoscopic prints in full colour. Nat. Commun..

[28] Jiang H, Kaminska B, Porras H, Raymond M, Kapus T (2019). Microlens arrays above interlaced plasmonic pixels for optical security devices with high-resolution multicolor motion effects. Adv. Opt. Mater..

[29] Jiang Q, Jin G, Cao L (2019). When metasurface meets hologram: principle and advances. Adv. Opt. Photon..

[30] Lim KTP, Liu H, Liu Y, Yang JKW (2019). Holographic colour prints for enhanced optical security by combined phase and amplitude control. Nat. Commun..

[31] Hu Y (2019). 3d-integrated metasurfaces for full-colour holography. Light Sci. Appl..

