# Supplementary material for: Nanooptical elements for visual verification
Source: Sci Rep. 2021 Jan 28;11:2426. doi: 10.1038/s41598-021-81950-w (PMC7844036; doi:10.1038/s41598-021-81950-w)
Supplement: Supplementary file 1 — Supplementary Information 1. [file 41598_2021_81950_MOESM1_ESM.docx]

Supplementary Information for

**Nanooptical elements for visual verification**

**Alexander Goncharsky^1^, Anton Goncharsky^1^, Dmitry Melnik^2^ and Svyatoslav Durlevich^1^***

***^1^*** *Research Computer Center, M.V. Lomonosov Moscow State University, Leninskiye Gory, 1, building 4, Moscow 119991, Russia*

***^2^*** *Computer Holography Centre Ltd., str.2, Proezd 4922, Zelenograd, Moscow, 124460, Russia*

**sdurlevich@ya.ru*

**Supplementary Video 1**: Video of the produced DOE (variant 1) taken when illuminated by a point source of white light. When the position of the source of light shifts, the observer may see kinematic effects of the motion of the two letters “A” and “B”.

**Supplementary Video 2**: Video of the produced DOE (variant 3) taken when illuminated by a point source of white light. When the position of the source of light shifts, the observer may see kinematic effects of the motion of the three letters “A”, “B” and “C”.

**Supplementary Video 3**: Video of the produced DOE with an hourglass image taken when illuminated by a point source of white light. When the position of the source of light shifts, the observer may see kinematic effect of the transformation of the letter “O” to the letter “K” while flowing from top to bottom of the hourglass.
